# Academic bedside rounding in 10 As: the decalogue technique

**DOI:** 10.1590/acb410526

**Published:** 2026-02-06

**Authors:** Diego Adão, Gabriela Caetano Lopes Martins, Leonardo Yuri Kasputis Zanini, Mateus de Almeida Oliveira, Parisina Fraga Carvalho, Leonardo de Mello Del Grande, Fernando Augusto Mardiros Herbella

**Affiliations:** 1 Universidade Federal de São Paulo – Escola Paulista de Medicina – Departamento de Cirurgia – São Paulo (SP) – Brazil.

**Keywords:** Education, Medical, General Surgery, Internship and Residency

## Abstract

**Purpose:**

To propose the 10 As decalogue technique, a structured framework for surgical bedside rounding.

**Methods:**

This narrative review and perspective piece presents a descriptive model for bedside rounding, designed for implementation in university hospitals. Literature review was done in MEDLINE via PubMed up to May 2025, using both free terms and Medical Subject Headings related to medical education, clinical rounds, and surgical training. The technique was developed based on clinical and educational principles, addressing both surgical and academic needs.

**Results:**

The decalogue consists of the following steps: anticipation, assistant team presentation, authorization, (self)appraisal, assessment, alerts, advice, agreement, annotation, and afterwards. It facilitates comprehensive case discussion, improves trainee clinical skills, and fosters empathy and trust in patient interactions. It addresses time constraints by prioritizing critical cases and enhances learning through debriefing and feedback. Challenges include managing time for multiple patients and ensuring patient comfort during public discussions, mitigated by clear authorization and sensitive communication.

**Conclusion:**

The 10 As decalogue is an effective tool for surgical bedside rounding, promoting resident education, reducing errors, and enhancing patient involvement. Its structured approach is particularly suited for academic settings, with adaptability to balance educational and clinical demands.

## Introduction

Sir William Osler said that “medicine is learned by the bedside and not in the classroom.” This principle is still valid after 100 years, even though it has been replaced, at least in part, by board or virtual style in current times^1^. Education of trainees depends on interacting with real patients (natural method). This experience is most useful when intermediated by an experienced instructor who focuses on relevant data and preserves the ethical principles of healthcare team-patient interaction^2^.

Structured teaching tools, such as the one-minute preceptor and the SNAPPS model, have been developed to enhance clinical education^3^^,^ 4. The one-minute preceptor is a concise teaching strategy based on five microskills, designed to optimize preceptor-student interactions^3^. The SNAPPS model, tailored for case presentations in outpatient settings, comprises six steps:

- Summarize briefly the history and findings;
- Narrow the differential to two or three relevant possibilities;
- Analyze the differential by comparing and contrasting the possibilities;
- Probe the preceptor by asking questions about uncertainties, difficulties, or alternative approaches;
- Plan management for the patient’s medical issues;
- Select a case-related issue for self-directed learning^4^.

However, the literature on bedside medical education remains limited. This study proposed the 10 As decalogue technique as a structured approach for surgical bedside rounding, aiming to enhance trainee learning, improve patient-team interactions, and ensure comprehensive, ethical patient care in academic hospital settings.

## Methods

We conducted a perspective piece and a narrative review of the literature. This narrative report details the authors’ experience utilizing the 10 As decalogue technique to a complete surgical bedside rounding. This technique was implemented in the surgical emergency department of a quaternary university hospital in an urban center in Brazil (Escola Paulista de Medicina, Universidade Federal de São Paulo) as of January 2024 to nowadays. Ethical approval was not required for this study, as no data was collected.

We conducted a narrative review of the literature using the MEDLINE database, accessed via PubMed, to identify relevant studies published up to May 2025. The search terms (free and Medical Subject Headings) included combinations of “education,” “residency,” “surgery,” “clinical skills,” and “surgical training,” using Boolean operators.

We excluded studies that were not in English. No strict date restrictions were applied, though emphasis was placed on recent literature to reflect current practices and insights. Articles were selected based on their relevance to the key themes of our review, which included medical education, clinical rounds, and surgical training.

The 10 As decalogue technique was designed to be implemented in university hospitals with a focus on resident and student education. It was developed based on clinical and educational principles, addressing both surgical and academic needs.

The impressions documented in this study reflect the perspectives of the preceptors and residents, who are also authors, based on their experiences with the decalogue and informal discussions with colleagues.

## Results

The 10 As decalogue consists of the steps described in Table 1:

**Table 1:** The decalogue 10 As technique for bedside academic rounding.

Principles	Example
Anticipation	Today we will begin our visit with patient X, who is hospitalized for treatment of disease Y.
Assistant team presentation	Hi. My name is Dr. A and I am the surgeon on the team that is taking care of you.
Authorization	We would like to discuss your case out loud. This discussion is intended to clarify doubts and teach academics. We will take care to avoid information that might embarrass you. Some things said may have nothing to do with your case. At the end, we will summarize everything that concerns you.
(Self)Appraisal	Please tell us what you know about your illness. What is the reason for your current hospital stay? What treatments have you been offered?
Assessment	Could you clarify for us some points in your medical history that were not clear? Could we examine your abdomen to certify the residents’ findings?
Alerts	Team member, please inform the nurse that this patient is allergic to medication X. Or notify the surgical team that this patient has peritonitis and needs to be re-evaluated.
Advise	After our evaluation, we agreed that you have disease X, whose most appropriate treatment would be Y. However, there are chances of it being disease A, whose treatment would be B. We will clarify and communicate as the facts become clear. Do you have any questions?
Agreement	We would like to know if, after knowing your diagnosis, treatment, and risks, you understand and agree with our therapeutic plan.
Annotation	Note in the medical record that we will start treatment for disease X and that the patient agrees with the treatment.
Afterwards	Does anyone have any questions about the case? I would like you all to study disease X through the article I›m going to send.

Source: Elaborated by the authors.

### Anticipation

A briefing must be carried out so that all participants are aware of the cases, containing the reason for hospitalization and possible treatment proposals. This leads to a better comprehension by the novice and allows the more experienced to interact and to express more meaningful opinions. Also, it prevents questions that may embarrass the patient and cause discomfort.

### Assistant team presentation

As the second step and first at bedside, the team must introduce themselves to the patient, sharing their name and role individually. In addition to making the team known, this presentation allows for closer ties, greater bonding, and empathy between the assistant team and patients. In cases in which the team is already known to the patient, as may occur in a longer hospital stay, the presentation may be brief or even excluded.

### Authorization

The senior must acquire patient’s authorization to publicly discuss the case among team members. Academic and care objectives must be explained. It is essential to clarify that many things said do not necessarily correlate with the case, and that privacy will be preserved. It is necessary to make it clear to the patient that, during the discussion phase, some questions may not have answers, or some answers by the trainers may be corrected by the staff, which does not mean disqualification of team members. At this point, at the end, a summary will be made with real data relevant to that patient.

### (Self)Appraisal

Patients should be questioned about comprehension of the disease, reason for hospitalization, and treatment already carried out, before clarifying the current situation. The situation can be used to assess the patient’s real knowledge and expectations, or even identify possible gaps and errors in communication. In some cases, family members may be present. It is essential to be aware of whether there is any confidentiality requested by the patient regarding the family. Family members may contribute with relevant information about the case.

### Assessment

Considering that the case is already known to the team members prior to the bedside visit, the discussion begins with questions aimed at possible doubts and gaps. Clinical history and physical examination are complemented. Preceptors can perform propaedeutic maneuvers to demonstrate or validate the findings made by the trainers.

### Alerts

Red flags may be identified during the round, such as clinical signs of hemodynamic instability, decompensation of underlying disease, dysfunction of devices such as probes or drains, need for surgery or identification of allergies. An alert must be issued to the responsible team, if not the one rounding, or immediate actions taken by the rounding team.

### Advise

One of the team members (ideally the most experienced or the person responsible for the case) explains to the patient the current state of health, certainty of the diagnoses, diagnostic hypotheses, differential diagnoses, therapeutic plan, and possible future scenarios. The language must be accessible and empathetic, respecting the limits of each patient’s understanding. Doubts may arise and these should be answered at this time. It is essential that in this moment bad news are avoided. Bad news must be reserved for another time and another communication strategy according to specific guidelines (e.g., Spikes)^5^. At this point, the preceptor can also demonstrate communication skills and play the role model.

### Agreement

Patients are asked about agreement with the team’s conduct and are offered involvement in the shared decision of the case. Informed consent for possible procedures can be acquired at this moment.

### Annotation

All decisions made during the visit must be annotated in the medical record stating that the full team was present to share responsibilities, as well as any intervention carried out, changes in treatment and whether the patient agreed with the treatment chosen.

### Afterwards

After the end of the round, the team, but specially the trainees, can be debriefed to clarify their doubts about the case, questions, and other explanations. The team leader can provide brief feedback on the members’ performance in the round and indicate complementary literature for study related to the case.

This topic aims to facilitate the learning of residents and students, with the goal of improving performance in future rounds and storing knowledge about the addressed pathology.

## Discussion

The bedside visit technique aims to better understand the case by the healthcare team, in addition to providing greater learning and clinical practice to trainees, which was compromised during the COVID-19 pandemic, when telemedicine visits prevailed^6^. Another important point is the strengthening of the doctor-patient relationship, which is enhanced by bedside visits^7^.

This strategy is valid for both clinical and surgical patients, and it is aimed at university hospitals where the focus is on resident and academic learning. However, even though the focus is on resident education, compared to other similar educational models, such as the one-minute preceptor or SNAPPS, the decalogue technique factors in patient, preceptor, and resident^3^^,^ 4. The patient participation can be perceived from step 2 (assistant team presentation) to step 8 (agreement). That is a different approach than proposed by Kogan et al., for example^8^. The authors proposed lists of recommended practices, contraindications, and uncertainties regarding direct observation as a method for evaluating clinical abilities in medical training. Nevertheless, patient’s comprehension of his/her clinical case and agreement to treatment plan are not given due regard.

A negative point is the time available to meet each patient overloading the team with several patients to discuss. A recommendation is that on busier days, a more objective approach is taken, prioritizing more important goals, and avoiding long unproductive discussions. One method is to start the round with patients who are new, still waiting for a definition of management or with those who are more seriously ill. This allows team members to make important decisions earlier, thus expediting the resolution of pending issues.

After a few days of rounds, the team leader must provide constructive feedback to the team members, evaluating the rounds and the individual performance of each member, aiming for improvement in the coming days.

Our study has several limitations, primarily due to its nature as a perspective piece. As no empirical data were collected, this paper reflects the authors’ subjective perceptions of the 10 As decalogue technique. Additionally, implementation challenges, such as resource variability (e.g., time, training, infrastructure) and institutional constraints, may limit scalability and generalizability. Future studies are needed to validate the technique and compare it with other educational tools.

## Conclusion

The 10 As decalogue technique is a tool for bedside visits that covers relevant topics. This promising approach warrants further formal evaluation to assess its potential to reduce errors and enhance engagement among patients, medical teams, and staff.

## Data Availability

Data sharing is not applicable.
